# What Is the Correct Answer about The Dress’ Colors? Investigating the Relation between Optimism, Previous Experience, and Answerability

**DOI:** 10.3389/fpsyg.2016.01808

**Published:** 2016-11-23

**Authors:** Bodil S. A. Karlsson, Carl Martin Allwood

**Affiliations:** Department of Psychology, University of GothenburgGothenburg, Sweden

**Keywords:** answerability, belief, judgment, color perception, optimism, non-believed perceptions, non-believed memories, The Dress

## Abstract

The Dress photograph, first displayed on the internet in 2015, revealed stunning individual differences in color perception. The aim of this study was to investigate if lay-persons believed that the question about The Dress colors was answerable. Past research has found that optimism is related to judgments of how answerable knowledge questions with controversial answers are (Karlsson et al., 2016). Furthermore, familiarity with a question can create a feeling of knowing the answer (Reder and Ritter, 1992). Building on these findings, 186 participants saw the photo of The Dress and were asked about the correct answer to the question about The Dress’ colors (“*blue and black,” “white and gold,” “other, namely…,”* or *“there is no correct answer”*). Choice of the alternative *“there is no correct answer”* was interpreted as believing the question was not answerable. This answer was chosen more often by optimists and by people who reported they had not seen The Dress before. We also found that among participants who had seen The Dress photo before, 19%, perceived The Dress as *“white and gold”* but believed that the correct answer was *“blue and black*.” This, in analogy to previous findings about non-believed memories (Scoboria and Pascal, 2016), shows that people sometimes do not believe the colors they have perceived are correct. Our results suggest that individual differences related to optimism and previous experience may contribute to if the judgment of the individual perception of a photograph is enough to serve as a decision basis for valid conclusions about colors. Further research about color judgments under ambiguous circumstances could benefit from separating individual *perceptual experience* from *beliefs about the correct answer* to the color question. Including the option *“there is no correct answer*” may also be beneficial.

## Introduction

**FIGURE 1:**
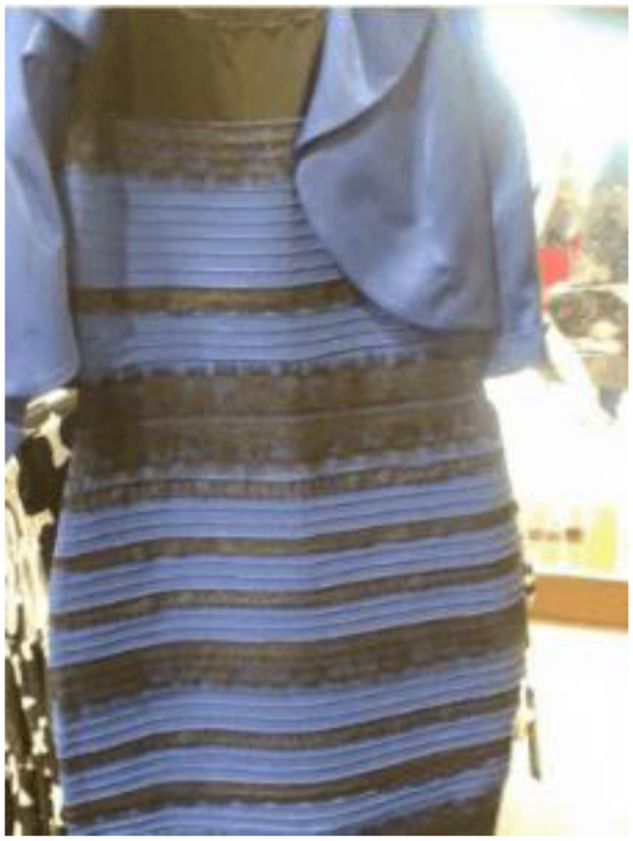
**The original photo of the dress.** Dress photograph reproduced with permission from the creator Cecilia Bleasdale (Dressgate, 2016).

The viral phenomenon The Dress revealed astonishing individual differences in color perception (Gegenfurtner et al., 2015). Some people claimed they saw a blue and black dress while others thought the dress was white and gold, referring to the same photograph. The quest to identify the “correct answer” about the colors of The Dress became a hot topic in social media (e.g., Akbaerian, 2015) and millions of people shared the photograph, among others, in order to make sense of it (see **Figure 1**). In this study we, in addition to asking about the participant’s perceptual experience, asked about what they believed was the correct answer, or if they believed *“there is no correct answer.”* We also investigated how individual differences in optimism and previous experience of The Dress photograph were related to beliefs that the question about the “correct colors” was answerable.

Before reviewing previous research on The Dress, it deserves to be noted that color perception and beliefs about color perception can be influenced by other information than the perceived object as such. For example, people often assign typical colors to objects, e.g., “bananas are yellow,” “my house is brown,” and the “Coca-Cola logotype is white and red,” despite the fact that physical stimuli may be ambiguous. These assigned typical colors can be considered socially prevalent knowledge and socially *correct answers* even though objects are not always *perceived* in their typical color and despite the fact that the *perceptual experience* that objects appear to have a constant color is created by the brain (Brainard et al., 2006). For example, a parent may ask a child “What color is the sun?” and expect “yellow” as the correct answer, even though the sun for example could appear red at sunset. It is possible that people may assign typical colors that they consider *the correct answer* to The Dress’ colors, despite the astonishing differences in *perceptual experience* of the photograph.

### Previous Research on the Colors of The Dress

Previous research has investigated the *perceptual experience* of The Dress’ colors (Brainard and Hurlbert, 2015; Gegenfurtner et al., 2015; Lafer-Sousa et al., 2015; Schlaffke et al., 2015; Winkler et al., 2015; Chetverikov and Ivanchei, 2016; Moccia et al., 2016). This research has suggested that individual differences in the perceptual experience of the colors of The Dress can be explained by differences in assumed light conditions in the scene when the photograph was taken (Gegenfurtner et al., 2015; Lafer-Sousa et al., 2015; Chetverikov and Ivanchei, 2016). For example, objects in shadow appear bluish, and the light blue pixels in The Dress photograph may be interpreted (unconsciously) as a white fabric that appears bluish because it is in the shade. The blue pixels can also be thought of as something blue presented in bright light (Gegenfurtner et al., 2015; Winkler et al., 2015). Different reasons for why some people are inclined to assume a specific light condition have been suggested.

One reason could be available cognitive capacity. Seeing white and gold is associated with higher brain activity (Schlaffke et al., 2015) and Moccia et al. (2016) found that people with reduced cognitive capacity due to Multiple Sclerosis were less likely to perceive the more demanding *“white and gold*.”

Another reason could be previous experience. Lafer-Sousa et al. (2015) argued that it could be because a person may be more used to certain light conditions (for example by being a morning-person or an evening person). Furthermore, Lafer-Sousa et al. (2015) found that participants with experience of the photograph used the terms *“blue and black”* and *“white and gold”* more, as opposed to other color terms. Accordingly, previous experience may be related to entertaining the belief that there is a *correct answer* to the color question.

This paper contributes to research about The Dress’ colors by asking for *beliefs about the correct answer*, including the answer alternative that the answer cannot be found. In addition, our study is the first to investigate if individual differences in optimism and previous experience of The Dress are related to judgments about if the question concerning The Dress’ colors is *answerable*.

### Previous Research on Answerability Judgments

Generally speaking, answerability judgments pertain to the issue of whether a question is considered to have a correct answer today, and if not, if it can be answered in the future. By a question being answerable, we mean that “a correct, well-argued, answer at a relevant granularity level can be provided to the question” (Allwood et al., 2016, p. 40). Research has only investigated judgments about the answerability of general knowledge questions (Allwood et al., 2016; Karlsson et al., 2016; Buratti et al., in press). Answerability judgments can also include the belief that a question, due to its nature can never be answered. When a group of participants were asked *when* a question could be answered (now, in a year, in 2 years, in 10 years, etc.,.…or never) these ratings were strongly correlated to *how likely* another group thought it was that someone could answer the question *today* (Karlsson et al., 2016). Answerability judgments are important for decision makers, for example because these judgments affect if additional information should be searched for in order to answer the question correctly, or if the question is already answered with the current information available.

It has been found that individual differences are related to judgments of answerability of difficult general knowledge questions, especially when there is a lack of socially prevalent knowledge about the answer or beliefs about answerability (Karlsson et al., 2016; Buratti et al., in press). For example, people with a high belief in certainty of knowledge are more prone to believe that someone can answer difficult questions today, or at least in the near future (Karlsson et al., 2016; Buratti et al., in press). However, as noted above, previous research on individual differences in answerability has only used general knowledge questions (e.g., in physics, grammar, geography, history) as stimuli. Ours is the first study investigating answerability of color judgments in general and The Dress in particular.

#### Optimism

Karlsson et al. (2016) found that level of optimism was correlated to lower judgments of the answerability today of difficult knowledge questions. The researchers suggested several explanations to why optimists are more likely to believe that no correct answer exists. For example, optimists expect good things to happen in uncertain times (Monzani et al., 2014) which could lead to that they accept and tolerate uncertainty in the environment more than others, such as questions without correct answers. Furthermore, optimists are known to try harder and be less likely to give up goals (Muhonen and Torkelson, 2005; Carver et al., 2009). For a difficult question, this could mean that optimists may spend more time and effort to think about different ways to answer and interpret the question. Since optimists in addition are less likely to use denial/distancing as strategy (Scheier et al., 1986), they may also be more prone to include diversity in their judgments which may lead them to conclude that *“there is no correct answer.”* Since judging the correct answer about the colors of The Dress can be considered difficult and optimists have been found to be more prone to judge difficult knowledge questions as not possible to answer today (Karlsson et al., 2016), optimists may to a larger extent believe that *“there is no correct answer”* to the question about the colors of The Dress.

#### Previous Experience

Another factor that may influence answerability judgments is previous experience of the question. People who report to have seen The Dress photograph before are more prone to use the terms *“blue and black,”* and *“white and gold”* (Lafer-Sousa et al., 2015) when judging The Dress’ colors and can be assumed to be more familiar with the question terms. Reder and Ritter (1992) found that familiarity with the question terms, rather than familiarity with the answer, determined the feeling of knowing the answer to the question. This familiarity with the question terms may increase the feeling of knowing the answer (Reder and Ritter, 1992), implying that there is an answer to the question, as opposed to the belief that no correct answer exists.

### Hypotheses

Our first hypothesis was that more optimistic people to a larger extent than less optimistic people would believe that *“there is no correct answer”* to the question “*What is the correct answer?”* about the Dress’ colors (*Hypothesis 1*). If this hypothesis is confirmed it would indicate that the relation between optimism and answerability judgments applies to other types of questions than the general knowledge questions used in previous research (Karlsson et al., 2016).

For reasons reviewed above, our second hypothesis was that people with previous experience of The Dress would be less likely to choose *“there is no correct answer”* to the question *“What is the correct answer?”* about The Dress’ colors, compared to people who have not seen The Dress before *(Hypothesis 2)*.

## Materials and Methods

### Participants and Design

In total 190 persons (137 female, 51 male, and 2 other) answered a web-survey. The mean age was 29 years, ranging from 18 to 61 years old. The present study followed ethical guidelines in Sweden for survey data. Participants were recruited from a pool of adults that had already actively volunteered and signed up for participation in psychological research. Data was collected in the context of an unrelated web-study and the time window was 1 week in the beginning of October 2015. Four participants were excluded since they did not provide complete answers to the questions about The Dress, leaving 186 participants to be further reported.

### Materials

#### Questions about The Dress

Three questions were asked about The Dress in the following order: *“Have you seen the dress before?”* (*“yes”; “no”; “don’t know”*), *“What colors do you perceive the dress to have?”* (*“blue and black”; “white and gold; “other, namely….”*) and *“What is the correct answer?”* (*“blue and black”; “white and gold”; “other, namely…”; “there is no correct answer”*).

#### Optimism

Personal optimism was measured with LOT-r which has six items (Monzani et al., 2014). An example of an item is “*In uncertain times, I usually expect the best*.” The items were answered on a five-point scale ranging from 1 = *“Do not agree”* to 5 = *“Totally agree*.” The Swedish translation of the scale was taken from Muhonen and Torkelson (2005). Cronbach’s alpha was 0.76.

### Procedure

At the end of an unrelated study performed in the participant pool, participants were asked if they would like to participate in another study in psychology and were provided with a survey link. All participants were informed that they had the right to end their participation at any time, that participation was anonymous, and that the data would be treated confidentially and only for research purposes. They agreed to participate by clicking the survey link.

After agreeing to participate in the web-survey, participants were exposed to The Dress photograph. While viewing the photograph they first answered two questions about The Dress mentioned above: *“Have you seen the dress before?”* and *“What colors do you perceive the dress to have?*” On the next page, participants were asked the third question about The Dress “*What is the correct answer?”* After the question about the correct answer, there was a possibility to leave a free text comment. Last in the session participants answered the LOT-r scale on personal optimism. Finally, they answered questions about gender and age. It was not possible to go back to previous pages.

## Results

**Table 1** shows, in cross-tabulated form, the participants’ answers to the three questions: *“Have you seen the dress before?*,” *“What colors do you perceive the dress to have?,”* and “*What is the correct answer?”* As can be seen in the table, of the 186 participants, 150 reported they *had* seen The Dress before, 33 claimed they *had not*, and 3 that they did not know. Moreover, 76 participants in total, chose that *“there is no correct answer”* to the question about the correct color.

**Table 1:** *Cross-tabulation of the Number of Answers to the Three Questions* “Have you seen the dress before?*” (Seen/Not seen)*, “What colors do you perceive the dress to have?” *(Perceptual Experience) and* “What is the correct answer?*”*

		“What is the Correct answer”				Total
		“Blue and black”	“White and gold”	“Other namely…”	“There is no correct answer”	
**Seen/not seen**	**Perceptual experience**					
“Seen”	Blue/Black	40	5	5	16	66
	White/Gold	28	8	1	29	66
	Other	4	3	2	9	18
	Total	72	16	8	54	150
“Not seen”	Blue/Black	6	0	0	7	13
	White/Gold	0	7	0	5	12
	Other	0	0	1	7	8
	Total	6	7	1	19	33
“Don’t know”	Blue/Black	0	0	0	1	1
	White/Gold	0	0	0	0	0
	Other	0	0	0	2	2
	Total	0	0	0	3	3
**Total**	Blue/Black	46	5	5	24	80
	White/Gold	28	15	1	34	78
	Other	4	3	3	18	28
**Total**		78	23	9	76	186

We used a multi-nominal logistic analysis to test if optimism and previous experience contributed significantly to the likelihood of choosing *“no correct answer*.” Dependent variable was *belief about the correct answer* (“*blue and black”; “white and gold”; others, namely…*.^1^; or *“no correct answer”*). Independent variables were optimism, previous experience of the photograph and perceived color. The reference category was *“blue and black*.” The model was significant, χ^2^(12) = 49.26, *p* < 0.001, Nagelkerke = 0.26 and all the independent variables contributed significantly: optimism, χ^2^(3) = 10.75, *p* = 0.013, previous experience, χ^2^(3) = 11.74, *p* = 0.008, and perceptual experience, χ^2^(3) = 26.71, *p* < 0.001.

**FIGURE 2:**
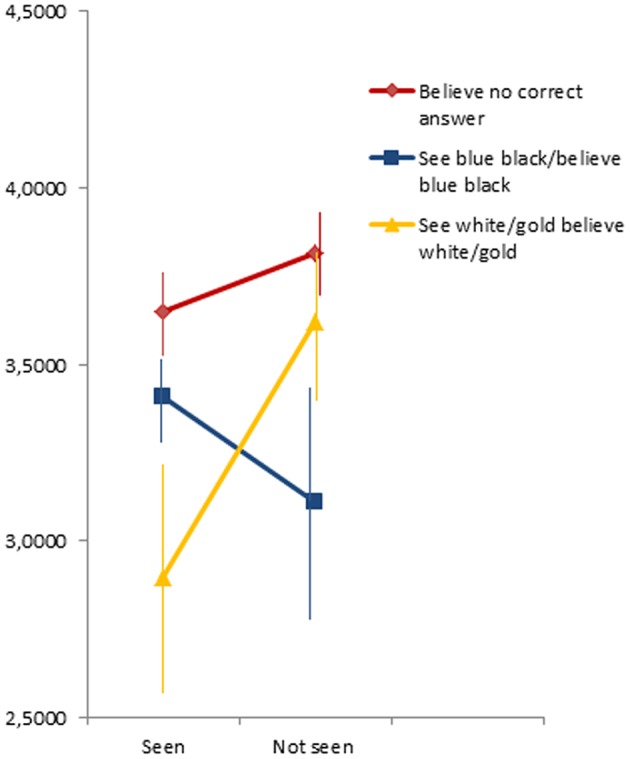
**Mean optimism scores, with error bars (standard error), for participants who had seen, or not seen, The Dress before and either believed *“there is no correct answer”* or showed consistency between their perception (*“What colors do you perceive the dress to have?”*) and their belief (“*What is the correct answer?”*) about The Dress’ colors.** These last participants are, for comparison, split up into participants who consistently choose either *“blue and black”* or *“white and gold.”*

**FIGURE 3:**
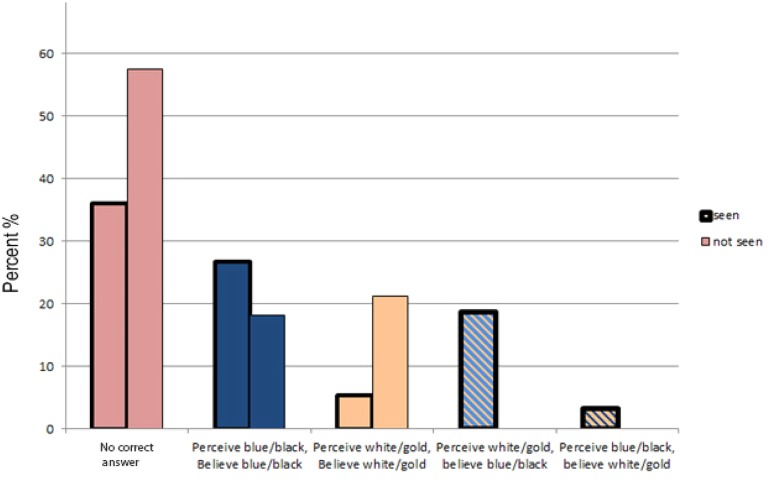
**Comparison between participants who had seen, and not seen, The Dress photograph.** The percentage of participants choosing *“no correct answer”* is compared to different combinations of perception and belief. The two left-most staples in this figure show the percentage of participants who choose *“no correct answer”* on the question *“What is the correct answer?”* among those who had seen and not seen The Dress photograph, respectively. The other staples show combinations of perceptual experience *(“What colors do you perceive the dress to have?”)* and beliefs about the correct answer *(“What is the correct answer*?”)^2^ among those who had seen and not seen the photograph, respectively.

As illustrated in **Figure 2**, higher optimism was significantly related to a higher probability of choosing *“there is no correct answer”* (*B* = 0.49, *p* = 0.024). As shown in **Figure 3** and **Table 1**, participants who reported experience of The Dress photograph, were less likely to say *“there is no correct answer”* about the colors of The Dress (*B* = -1.37, *p* = 0.01). Participants perceiving *“blue and black”* were also significantly less likely to say *“there is no correct answer”* (*B* = -2.07, *p* = 0.001).

### Additional Findings

#### Optimism

The eight participants who had *seen* the photograph before and who saw and believed *“white and gold”* scored marginally significantly lower on optimism than the seven respondents who reported that they had *not seen* The Dress photograph and who reported to both perceive and believe *“white and gold*,” *F*(1,13) = 3.82, *p* = 0.073. The interaction between those who had seen and not seen the photograph, with consistent perception and belief of either *“white and gold”* or consistent perception and belief of “*blue and black,”* is illustrated in **Figure 2**, and was significant, *F*(1,57) = 5.38, *p* = 0.024. These results indicate that optimism was related to individual differences in belief-updating regarding the correctness of one’s own color perception.

#### Previous Experience

Among the 150 participants who had seen The Dress before, 31% (46 participants) reported to believe in a correct answer that did not match their own recent perceptual experience. Here, 28 participants (42%) of the 66 participants who saw The Dress as *“white and gold”* believed *“blue and black*” was the correct answer. Five (8%) of the 66 participants who perceived *“blue and black”* considered *“white and gold”* the right answer. As illustrated in **Figure 3**, the proportion of participants who chose *“blue and black”* to be the correct answer, in contrast to their own previously reported perception of *“white and gold,”* was larger than the proportion of the participants who chose *“white and gold”* to be the correct answer, in contrast to their own perception of *“blue and black”* (χ^2^ = 8.76, *p* = 0.03). Thus, non-believed previous perceptions of *“white and gold”* were more common than non-believed perceptions of *“blue and black*.”

In contrast, the 33 participants who had *not* seen The Dress before, either answered that the correct answer was the color they perceived (14 participants, 42%) or that there was no correct answer (19 participants, 58%). Thus, none of these 33 participants showed a mismatch between perceived and believed colors.

## Discussion

The main purpose of this study was to investigate lay persons’ beliefs about the answerability of the question about The Dress’ colors and how trait optimism and previous experience were related to the belief that a correct answer about The Dress’ colors exist, that is, if the question is considered answerable. The results showed that as many as 41% of the participants choose the option *“there is no correct answer*.” Below, we first discuss the results for optimism, then for the effect of previous experience of the photo, followed by a discussion of the additional findings.

### Optimism

In line with our first hypothesis, the results showed that more optimistic participants tended to believe that “there is no correct answer” to the question about The Dress’ colors. Although not explicitly investigated in the present study, different factors may have contributed to this result. For example, it may be easier for optimists to accept that questions might not be answerable since optimists are more prone to think that something good might come about even in uncertain circumstances (Monzani et al., 2014). Moreover, previous research has shown that optimism correlates fairly highly with self-efficacy. Different studies show a correlation between optimism and self-efficacy between 0.2 and 0.6 in different countries (Luszczynska et al., 2005; Karademas, 2006). Given their greater self-perceived ability to deal with challenges, optimists may be better prepared and more willing to deal with uncertain situations, such as there being no correct answer. As reviewed above, previous research on general knowledge questions with no generally accepted answer, has reported that optimists believed these questions were less answerable (Karlsson et al., 2016). The present research contributes by indicating that this finding may generalize to judgments about color perception.

### Previous Experience

Our results show that participants who reported to have seen The Dress before significantly less often choose the option *“there is no correct answer*.” Thus, in our data-set previous experience of The Dress photograph was related to the belief that there is a correct answer. This finding may be explained in several ways, and below we, speculatively, suggest three that may contribute together or separately.

#### Familiarity

The first explanation is, as described in the Introduction, the effect of familiarity with the question. Familiarity may increase the feeling of knowing the answer (Reder and Ritter, 1992), and consequently also the belief that the question is possible to answer.

#### Social Influence

The second explanation is that social influence can make people more likely to believe that there is a correct answer. Participants with previous experience of The Dress photograph are more likely to have knowledge about other people’s *perceptions* and *beliefs* about The Dress’ colors than participants who report they have not seen the photograph. Johansson and Allwood (2007) used general knowledge questions and found that their participants reported higher ratings of the extent of other’s knowledge and higher confidence in the correctness of others’ knowledge (a friend), compared to their ratings of their own knowledge. Accordingly, in cases where people are uncertain and listen to someone they trust, they may show an overconfident belief in the other person’s answers. For example, there may be a tendency for people with experience of The Dress photograph to be aware that people who have seen The Dress in real life tend to agree that The Dress is blue and black (e.g., Akbaerian, 2015). As a consequence they may believe that there is a correct answer for example based on how The Dress is typically perceived in real life, that is, blue and black.

#### Affirmation

A third explanation relates to affirmation. If a person makes the same judgment many times, they also seem to be more confident that the answer is correct (e.g., Hertwig et al., 1997; Knutsson et al., 2011; Koriat, 2011, 2012; Unkelbach et al., 2011). Thus, when making the *same perceptual judgment* several times (e.g., *“blue and black”*) people may convince themselves this must be the correct answer. Since the majority of participants do not seem to shift in perception of the colors of The Dress photograph from one occasion to another (Chetverikov and Ivanchei, 2016) previous experience can increase the likelihood of affirmation and believing in a correct answer due to this affirmation.

However, more research is needed in order to investigate under what circumstances, previous experience may lead to the belief that a correct answer exists since there are also reasons to believe that experience may lead to the conclusion *“there is no correct answer*.” For example, Munro (2010) found that people who have an opinion that contradicts the scientific consensus may conclude that the question cannot be answered within the frame of science. Thus, if a person initially has a belief that is in contrast to the majority of trusted others (e.g., *“white and gold”* in contrast to a majority of *“blue and black”*), they may conclude that the question is not possible to answer. Experience of the photograph may also be related to increased knowledge about the possible variation in *color perception* of the photograph. Awareness of the complexity of the judgment could possibly lead to that some people choose a more humble approach and conclude *“there is no correct answer.”* Irrespective of the specific mechanisms associating prior experience of The Dress with belief that a correct color answer exists, the results in the present study suggest that prior color experience of an object is related to belief that there is a correct answer to questions about the object’s color.

### Additional Findings: Non-believed Perceptions

Out of the participants who reported previous experience of The Dress, 28 participants (19%, 28/150) claimed *“blue and black”* was the *correct* answer, in contrast to their own recent perception of *“white and gold.”* Thus, when judging beliefs about the correct answer, some participants did not choose their own recent perception (that they had stated within a few seconds or minutes before) to be the correct answer. We call this phenomena “*non-believed perceptions*” since these people do not believe their recent perception is the correct answer to the question about the correct answer of The Dress colors.

In memory research it has been found that memories of events where people have no own recollection to rely on can be considered very plausible (for example the memory of a parent’s story about something that happened to a child). On the other hand, people can have own vivid recollections of visual detail, that they consider less plausible, and these memories may be non-believed (Scoboria and Pascal, 2016). Blank (2016) suggests that so-called non-believed memories (e.g., Mazzoni et al., 2010) can be part of a “healthy meta-cognitive reality check” that draws “on general world knowledge and external/social information.” Further research should explore if this also may be the case for perceived, but not believed, objects. In case of The Dress, participants who believe *“blue and black”* is the correct answer, despite recently seeing *“white and gold”* may refer to other people’s judgments. For example, The Dress is typically considered blue and black in real life (e.g., Akbaerian, 2015) and it is possible that memories of such knowledge can affect beliefs about the correct answer.

Furthermore, it is known that the memory of socially prevalent knowledge of typical object colors, such as “bananas are yellow” can modulate *perception* (Hansen et al., 2006). Bananas are for example perceived to be more yellow than their reflected light spectra conveys (Hansen et al., 2006).

Our data indicate that people with experience of The Dress photograph were less likely both to perceive and believe *“white and gold.”* Furthermore, participants seeing *“blue and black”* were less likely to report *“there is no correct answer.”* A possibility is that memories of socially prevalent knowledge that the typical color of The Dress is not *“white and gold”* may have guided both beliefs about the correct answer and the perception of The Dress. More research is needed in order to find out if such memories can alter perception of an artificial object such as The Dress. The memory “bananas are yellow” may be useful for survival and can help an individual to identify a banana in morning light were yellow objects may appear grayish (Hansen et al., 2006). Even though finding the right dress may not be a case of survival, believed memories such as “The Dress is typically blue and black” may help to identify The Dress in another context. Considering that colors in general is an important aspect of object recognition (Gevers and Smeulders, 1999) and that colors in the case of The Dress may be an important aspect in order to be able to identify a photographed garment in the future, it may not be unreasonable to do as 28 participants did in this study: to state *“blue and black”* as the correct answer, while seeing something else themselves. However, the phenomena that, we denote non-believed perceptions can have many explanations, and it should be further researched in order to be better understood.

### Limitations and Further Research

This study is limited in several ways and is an early attempt to apply the framework developed within research on answerability judgments of general knowledge questions (Allwood et al., 2016; Karlsson et al., 2016; Buratti et al., in press), on color judgments. This novel approach to color judgments opens up for new perspectives and research possibilities, but several limitations should be noted.

For example, our finding that participants seeing *“blue and black”* were less likely to report *“there is no correct answer”* suggest that *previous perceptual experiences* may be related to *answerability judgments*. However, in order to more reliably investigate the effect of *previous perceptions* on answerability judgments of The Dress photograph, more specific information about how many times, when and under what circumstances participants has seen the photograph, and if they shifted in perception, should be measured. Still, the importance of the issue of the specific amount of previous experience of The Dress picture is somewhat ameliorated by the research by Hertwig et al. (1997) who found that the reiteration effect (increase in confidence with repetition) is greatest between the first and the second repetition, thereafter the increase goes on but the increase is descending.

The character and amount of social influence that may be a part of previous experience in this study also needs to be further explored. Social influence can affect memories of colors (e.g., Loftus, 1977; Martin, 1997) which in turn could modulate future perception (Hansen et al., 2006) and both previous perceptions, and social influence (Allwood et al., 2016), may interact with answerability judgments.

Furthermore, as camera technology has developed and mobile camera devices are widely spread, many important decisions are, at least partly, based on photographs: The astronomer may use satellite photographs in order to judge if there is evidence for life on Mars; a medical doctor may judge the health of an unborn child based on ultrasound pictures; an ambiguous photograph, taken by a cell-phone, just like The Dress photograph, may be used as evidence in a crime case. This study investigated answerability judgments of “the correct colors” in a photograph where astonishing individual differences in color *perception* were found. The increasing spread of photographic technology in society contributes to making this topic increasingly important.

We found that *answerability judgments* of the “correct colors” differed greatly between individuals, and that these differences were statistically related to optimism and previous experience. However, given that our study is the first to investigate the judged answerability of questions about color perception more research is needed in order to confirm our results. Furthermore, other individual difference variables such as Locus of Control, Circadian Typology and Synesthesia may also be of interest to explore when it comes to color judgments in general and on color judgments based on photographic information in particular.

## Conclusion

This study suggests that a color judgment can be a decision process that includes *both individual perceptual experiences* and *beliefs* about colors. Even if these two phenomena are related they sometimes differ. We found that individual differences in optimism and previous experience were related to answerability judgments about the “correct answer” to the colors of The Dress. Higher optimism was associated with the belief that *“there is no correct answer”* and people with previous experience were less likely to choose *“there is no correct answer.”* Further research about color judgments under ambiguous circumstances could benefit from separating individual *perceptual experience* from *beliefs about the correct answer* to the color question. Including the option *“there is no correct answer*” may also be beneficial.

## Author Contributions

BK has had the main responsibility for data-collection and statistical analysis. BK and CMA have had shared responsibility for the remaining aspects of this research.

## Conflict of Interest Statement

The authors declare that the research was conducted in the absence of any commercial or financial relationships that could be construed as a potential conflict of interest.

## Footnotes

1 Participants choosing *“others, namely….”* on the question about the correct answer and participants choosing *“I don’t know”* on the question if they had seen The Dress before, were excluded from the multi-nominal regression since they were very few.

2 For reason of simplicity the few participants who choose “*others, namely…,”* and the participants *who did not know* if they had seen the photograph are not included in the graph. The percentage is based on the total amount of participants in each group and therefore the percentage for the seen and not seen groups, respectively, do not add up to 100% in the figure.
